# Chemical Interactions at the Interface of Au on Bi_2_Se_3_ Topological Insulator

**DOI:** 10.1021/acs.jpcc.4c04241

**Published:** 2024-09-13

**Authors:** Matjaz Valant, Sandra Gardonio, Saul Estandia, Mattia Fanetti, Andrey Vladimirovich Matetskiy, Polina Makarovna Sheverdyaeva, Paolo Moras, Vasiliki Tileli

**Affiliations:** † University of Nova Gorica, Vipavska 11, 5000 Nova Gorica, Slovenia; ‡ Institute of Materials, École Polytechnique Fédérale de Lausanne, CH-1015 Lausanne, Switzerland; § CNR-Istituto di Struttura Della Materia (CNR-ISM), SS 14, Km 163.5, 34149 Trieste, Italy

## Abstract

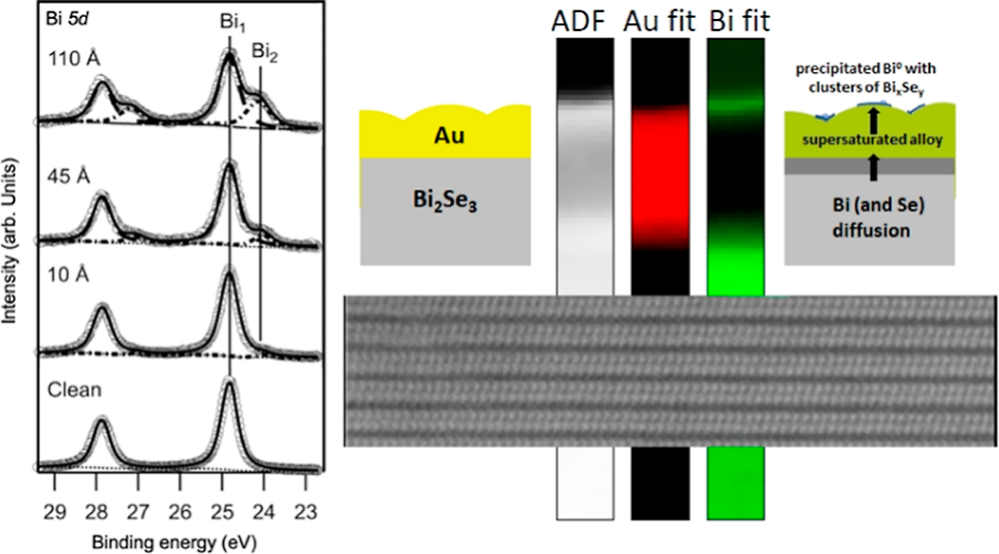

This study explores the intricate chemical processes at the interface between the topological insulator Bi_2_Se_3_ and deposited Au. The study mainly focused on room-temperature interactions that can cause the aging of, e.g., gold contacts on electronic devices based on the topological insulators (TIs) or spintronic devices. Our investigation uncovers a complex mechanism involving redox reactions, diffusion, and structural changes akin to the vapor–liquid–solid process. We observe the precipitation of metallic bismuth on the top of the Au layer and also a similar process, albeit at a slower rate, involving Se^0^. The resulting non-stoichiometry in the interfacial layers is compensated with the formation of an intermetallic compound low on Bi. As the temperature increases, Se diffusion intensifies, now leading to a selenium deficiency at the interfacial region and subsequent restructuring of the interface. These findings provide valuable insights crucial for optimizing material design and device performance, thereby guiding future research endeavors and technological advancements in the field of devices based on TIs.

## Introduction

Topological insulators (TIs) belong to a unique class of materials distinguished by the presence of a bulk band gap, which is akin to conventional insulators. However, their remarkable characteristic lies in the existence of protected conducting states residing along their edges or surfaces, known as topological surface states (TSSs). These distinctive states emerge due to the combination of spin–orbit coupling and time reversal symmetry. TSSs behave as Dirac electronic states because their energy-momentum *E*(*k*) relation is linear. Moreover, their spin is locked to the momentum, which results in spin-polarized transport on the surface/edge and significant suppression of backscattering by impurities and defects on the surface. Due to all of these unique sets of properties, these materials have generated significant interest in both fundamental physics and potential technological applications.

In quantum computing, TIs can serve as a platform for building topological qubits that are highly robust against local noise and perturbations. Their protected surface states can be used to encode and manipulate quantum information.^1,2^ TIs can be used in spintronic devices for the efficient generation, manipulation, and detection of spin-polarized electrons. This could lead to more energy-efficient and faster electronic devices.^2,3^ Researchers are exploring the possibility of using TIs to create topological field-effect transistors. These transistors can operate at lower power and higher speeds than traditional silicon-based transistors.^4,5^ Some TIs exhibit the quantum Hall effect without an external magnetic field. This property can be utilized in precision measurement devices, such as voltage standards and high-precision resistance measurements.^6,7^ TIs’ unique electronic properties make them suitable for various sensor applications, including gas and magnetic fields as well as for surface plasmon resonance sensors.^8−10^ In addition, TIs have potential applications in terahertz radiation generation and detection, which can be used in high-frequency communication and imaging systems.^11^

It is important to note that while TIs hold great promise, many of these applications are still in the experimental or theoretical stages. Further research and development are needed to realize their full potential.

One of the problems that researchers are facing is the right choice of the electrode material. The surface of 3D TIs, such as Bi_2_Se_3_ and Bi_2_Te_3_, is very reactive against metals, prone to redox reactions already at room temperature (RT). There are two primary consequences of this unwanted interaction to consider. First, the formation of new interfacial phases may influence the nature of the contact. For instance, it may affect the exploitation of TSS or favor the hybridization of the TSS with the contact layer and/or the newly formed interfacial layer, as recently reported in the case of Pd diffusion inducing polarization and superconductivity in a TI-based heterostructure.^12^ Second, as the reaction progresses, the quantity of quantum layers diminishes, potentially reducing the thickness of the TI to a level when the wave functions of the TSS on the top and bottom surfaces begin to overlap. This would result in a gap opening and the disappearance of the TSS. In particular, this is relevant for thin-film or nanostructured TI-based devices.^13^

The interfacial redox reaction has been observed in nearly all metals, including noble metals such as Ag and Cu.^14−16^ Some studies have attempted to mitigate this issue by applying a Ti or Cr barrier layer; however, subsequent research has demonstrated that these metals are even more susceptible to reacting with Au, leading to the formation of extensive reaction layers.^14^ Typically, the redox reaction produces metallic Bi and either binary or ternary chalcogenides with the electrode metal ions. So far, the least interaction has been shown for gold.^13^ Still, gold also seems to trigger some redox processes on the interface with Bi_2_Se_3_ as indicated by previous X-ray photoelectron spectroscopy (XPS) studies.^17^ Overlooking this aspect could lead to dubious or inaccurate interpretations of the optical and electronic properties of the Au–Bi_2_Se_3_ composite system.^18^ As the best (but not an ideal choice) electrodetectors for TI-based devices, Au and its chemical interaction with the TI surface must be fully understood. For that reason, we have undertaken a comprehensive study of the chemistry of this interface. We used XPS and transmission electron microscopy (TEM) techniques for the investigation of the system during aging at RT but also at elevated temperatures.

## Experimental Section

### Bi_2_Se_3_ Crystal Growth

High-quality single Bi_2_Se_3_ crystals were grown by the Bridgman method. Bismuth and selenium were purchased from Sigma-Aldrich. The stoichiometric amounts (Bi/Se = 2:3) of high-purity elements were sealed in evacuated quartz ampules and heated up to 750 °C at 21 °C/h. The ampules were maintained at that temperature for 48 h. Thereupon, the temperature was slowly reduced to 250 °C at 5 °C/h and then cooled down to RT.

### XPS Characterization and Analysis

Au depositions and XPS experiments were performed at the VUV-photoemission beamline of the Elettra synchrotron (Trieste, Italy). Clean surfaces of Bi_2_Se_3_ were obtained by in situ cleavage in an ultrahigh vacuum system with a base pressure of 2 × 10^–10^ mbar. Low energy electron diffraction was used to monitor the crystalline quality of the clean Bi_2_Se_3_(0001) surface. Various Au depositions (99.999% purity) were performed in situ using a well-outgassed, resistance-heated evaporator. The Au evaporation rate was calibrated by a quartz microbalance and cross-checked on a target W(110) sample, on which Au forms the (111)-like film with a sharp interface without chemical intermixing with the substrate. We used the thickness-dependence of the sp quantum well states of Au(111) films on W(110).^19^ As the quantum well state spectra are sensitive to thickness variations of one atomic layer [2.35 Å in Au(111) films], our accuracy can be estimated to be on the order of ±2 Å. During Au depositions, the Bi_2_Se_3_ samples were kept at RT. The XPS data were measured with a SCIENTA R4000 electron analyzer, which is placed at an angle of 45° with respect to the direction of the photon beam. The core level spectra were acquired with a 30° angular acceptance. The spectra were recorded in a normal emission geometry. All XPS spectra were recorded with a photon energy of 650 eV and a total energy resolution (electron spectrometer and monochromator) of ∼200 meV. All XPS measurements were performed at RT and a base pressure of less than 1.2 × 10^–10^ mbar. Se 3d and Bi 5d core level spectra were fitted with the Doniach–Sunjic function convolved with the Gaussian function on the Shirley background. The asymmetry parameter was set to 0. In the fitting, the spin–orbit splitting and the core-hole lifetime (Lorentzian width) were kept constant. For the Se 3d and Bi 5d core level components associated with Bi_2_Se_3_, a nonstatistical branching ratio had to be used. The deviation could be due to photoelectron diffraction effects. The intensity, energy, and Gaussian widths of the doublets were considered as free parameters.

### Electron Microscopy and Analysis

The cross sections of the gold film on Bi_2_Se_3_(0001) samples for the scanning TEM with electron energy-loss spectroscopy (STEM/EELS) analyses were prepared by depositing a 20 nm (nominal) thick Au film in a Precision Etching and Coating System (PECS, Gatan) with base pressure ∼2 × 10^–6^ mbar. The deposition was performed on Bi_2_Se_3_ single crystals, which were placed in a vacuum immediately after cleavage, with a deposition rate of 0.5 Å/s (measured during deposition with a quartz microbalance). The lamellas were obtained with a Zeiss NVision focused ion beam instrument and the final thinning of the lamellae was performed at 5 kV and 30 pA. STEM and EELS data were acquired on a probe-corrected ThermoFisher Scientific Titan Themis 60–300 equipped with a Gatan Quantum spectrometer. Experiments were performed at 300 kV with a probe current of 100 pA. For the EELS measurements, the convergence semiangle was 20 mrad and the collection semiangle was 94 mrad. The analysis of the EELS elemental maps was done with the Gatan Microscope Suite software and included background subtraction, multiple linear-square fitting using standard spectra, and integration of the signal of each edge component for each pixel. For the heating experiments, the DENSsolutions Wildfire TEM holder was used and the samples were prepared in heating chips (as shown in Figure S1).

## Results and Discussion

The initial phase of our investigation focused on the room-temperature processes occurring at the interface of Bi_2_Se_3_ and deposited Au. In a previous work,^17^ we observed that Au deposited at RT in the coverage range from <2 Å up to 110 Å, forms 3D island following the Volmer–Weber growth mode. We have already unveiled some degree of the interaction. A network of 3D coalesced islands, which was unable to completely cover the substrate, was observed at 110 Å of Au. Using XPS on this non-continuous film, it was not possible to exactly determine the location of the precipitated metal bismuth and trace the origin of the newly observed faint selenium component. This prevented us from comprehensively explaining the diffusion mechanisms at play. To gain a more thorough understanding of these processes, we have expanded our XPS analysis to include samples with significantly higher nominal coverages of Au (400 and 1090 Å) to ensure the complete and continuous coverage of the Bi_2_Se_3_ surface.

**Figure 1:**
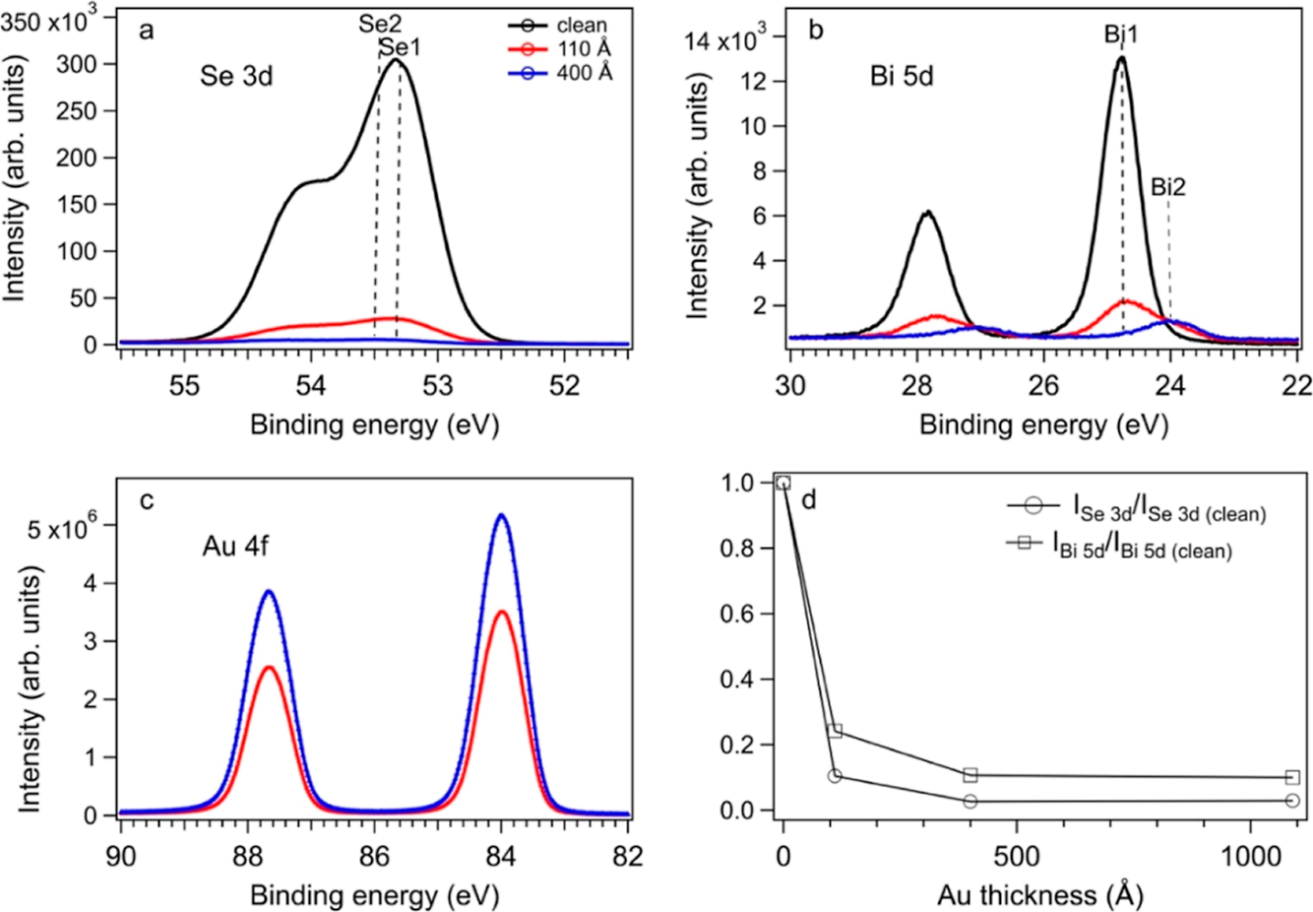
(a) Se 3d, (b) Bi 5d, and (c) Au 4f core level spectra measured on a clean Bi_2_Se_3_(0001) surface and after coating the surface with 110 and 400 Å thick gold film. (d) Ratio between the background-subtracted area of the Bi 5d and Se 3d core level spectra measured at different gold thicknesses with respect to the background-subtracted area of the Bi 5d and Se 3d measured on clean Bi_2_Se_3_(0001). The Se 3d_5/2_ and Bi 5d_5/2_ peak BE of the fitting components Se1, Se2, Bi1, and Bi2 shown in Figure 2 are indicated in (a) and (b).

After the initial depositions of gold, the signal intensity of the Bi 5d and Se 3d core levels decreased and then stabilized at nominal coverages of 400 Å. It remained constant up to 1090 Å. In this thickness range, the background-subtracted area of the Bi 5d core level accounted for about 10% of that observed on pristine Bi_2_Se_3_(0001), whereas the background-subtracted area of the Se 3d core level was only about 3% of that on the clean Bi_2_Se_3_(0001) (Figure 1). With thickness in the range 400–1090 Å, the Au film is compact and covers the entire substrate. The weak intensity of the Se 3d and Bi 5d core level spectra can be attributed to a small amount of selenium and bismuth on the surface of the gold film. For the entire range of Au coverage up to 1090 Å, the Au 4f spectra correspond to metallic gold [i.e., the binding energy (BE) of Au 4f_7/2_ is at 84.00 eV].

**Figure 2:**
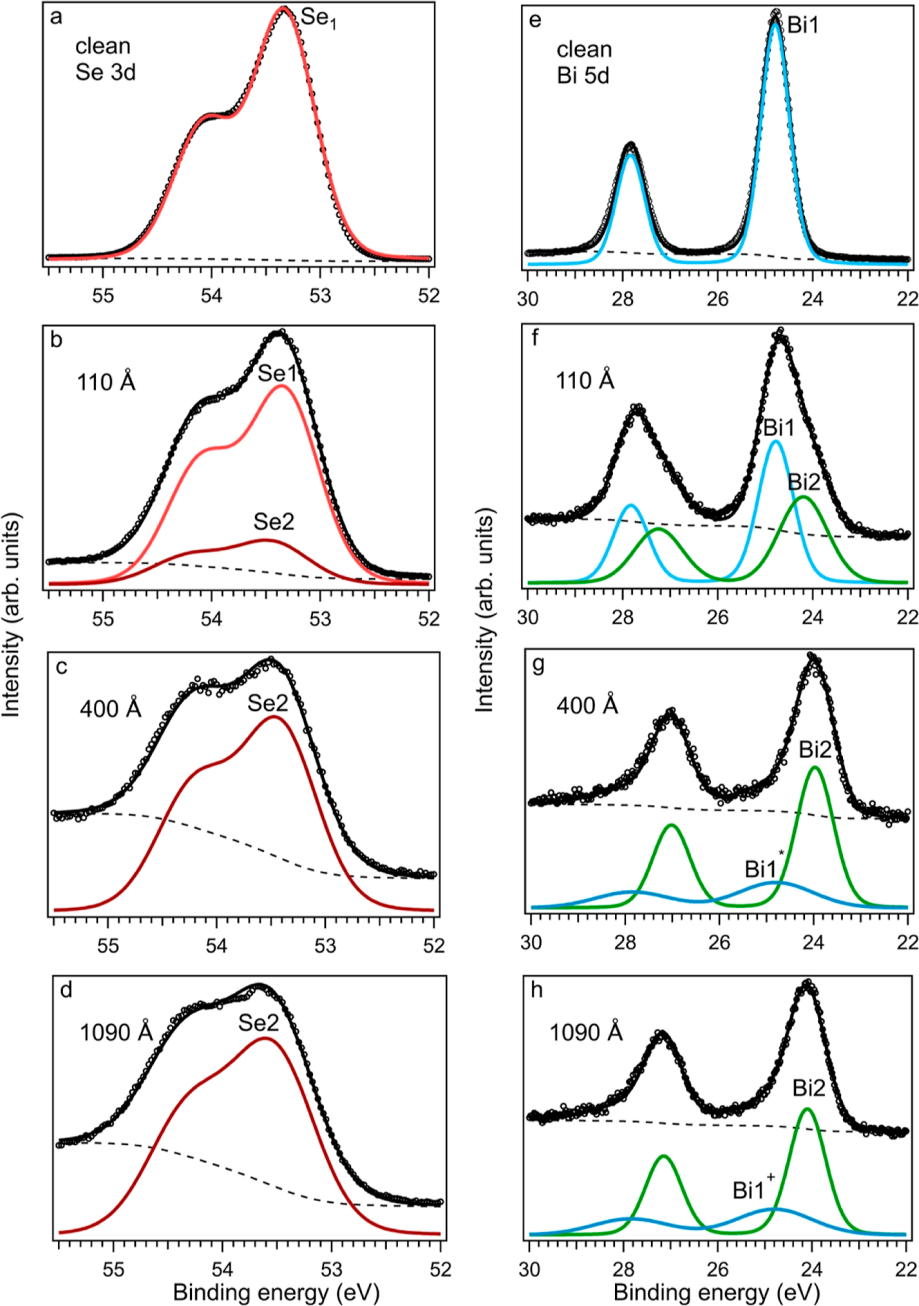
Core level spectra of (a–d) Se 3d and (e–h) Bi 5d measured on clean Bi_2_Se_3_(0001) and after coating the surface with a gold layer of a thickness of 110, 400, and 1090 Å. The results of the fitting procedure (see the Experimental Section) are also reported. Open circles—experimental data, solid line—best-fit results, and long dashed line—Shirley background. The colored lines are the fitting components Se1 (light red), Se2 (dark red), Bi1 (light blue), Bi1* (dark blue), and Bi2 (green).

The spectra of the Bi 5d core levels can be decomposed into two components (see Figure 2): Bi1 corresponds to Bi^3+^ with Bi 5d_5/2_ at 24.70 eV BE and Bi2 with Bi 5d_5/2_ at 24.00 eV BE corresponding to metallic bismuth. The spectra of Se 3d core levels can be deconvolved into two components: Se1 (light red) corresponding to Se^2–^ with Se 3d_5/2_ at 53.33 eV BE and Se2 (dark red) with Se 3d_5/2_ between 53.42 and 53.53 eV BE that can be attributed to selenium atoms with a lower electron charge than Se^2–^.^16^ By increasing the gold film thickness, the components Se1 and Bi1 decrease with respect to Se2 and Bi2.

These findings provide strong evidence for the presence of metallic bismuth on the surface of relatively thick and compact Au films. It is highly improbable that the observed Bi signal originates from bismuth alloyed with the Au throughout the bulk of the gold film, as its solid solubility limit, determined to be < 0.04 at %,^20,21^ falls well below the sensitivity threshold of XPS. The Bidiffusion process is accompanied by a very weak diffusion of selenium from the substrate. At the top of the Au film, selenium likely forms Bi_*x*_Se_*y*_ clusters with the surface-confined bismuth. This hypothesis is supported by the persistence of the Se2 (dark red) and Bi1*(dark blue) components (see Figure 2) even after depositing 400 and 1090 Å of gold onto the Bi_2_Se_3_ surface. Bi1* component has the same BE as Bi1. In this thickness range, the Bi1* and Se2 components increase their Gaussian broadening compared to Bi1 and Se1 of clean Bi_2_Se_3_, which is why they are marked in dark blue and red in Figure 2. The larger width of Bi1* and Se2 could be due to the actual existence of high structural and chemical disorder. The larger width of Bi1* and the small BE shift (∼150 meV higher BE) of Se2 with respect to Se1, could be due to the formation of clusters of the Bi_*x*_Se_*y*_ phase for which the chemical environments of bismuth and selenium slightly differ from that of Bi_2_Se_3_. The BE of Se2 is also consistent with Se chemisorbed on Au,^22^ indicating the possibility that some Se atoms are also bound to the gold surface.

**Figure 3:**
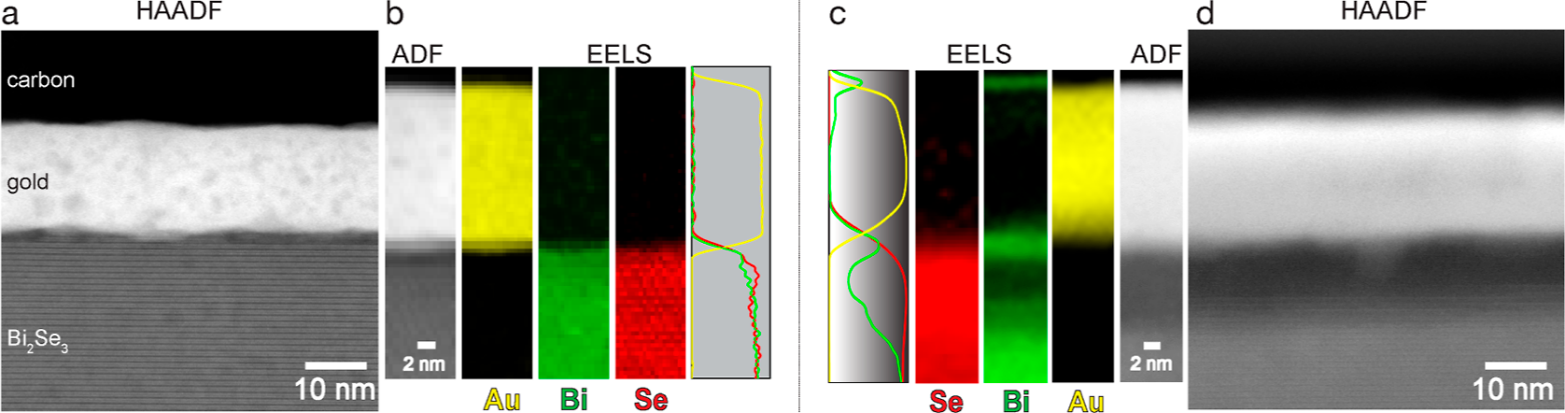
STEM/EELS analysis of the effects of the gold layer on the Bi_2_Se_3_ along the [1000] zone axis. (a) HAADF/STEM of thin Au/Bi_2_Se_3_ interfacial and (b) ADF image, elemental maps of Bi–M (green), Au–M (yellow), Se–L (red) edges, and relative quantification profile across the Au–Bi_2_Se_3_ interface. (c,d) Same as in (a,b) but for the region exhibiting a thicker interface and stronger chemical reactivity.

The local chemical environment between the Au film and Bi_2_Se_3_ was investigated using STEM-EELS on a sample in which the thickness of the Au film was approximately 20 nm. Several lamellae were analyzed from the same Au/Bi_2_Se_3_ sample, and the results showed that there was some heterogeneity across the sample, with areas of two distinct interfacial characteristics identified. Figure 3 shows the cross-sectional high-angle annular dark field (HAADF) STEM images and EELS analysis of the two regions. The gold layer (appearing bright) sits conformally on the Bi_2_Se_3_(0001). The upper gold surface was coated with carbon (appearing dark) to protect it during preparation. In both cases, a darker interface region is seen in the HAADF/STEM images but with significantly different thicknesses when comparing Figure 3a,d. The EELS relative quantification of the thin interface, Figure 3b, shows that a sharp interface is formed between Au and Bi_2_Se_3_ and that there is no chemical intermixing with constant Bi and Se components up to the bottom Au interface. In parallel, there are regions where EELS analysis clearly identified a stronger chemical interaction between Au and Bi_2_Se_3_, which is also associated with chemical features at the top of the Au interface. This interaction is characterized by an extended interfacial region, as seen by the darker contrast in the HAADF image, which is about 10 nm thick (Figure 3d). The EELS elemental maps (Figure 3c) reveal that while Se remains unchanged at this interface, the Bi concentration decreases within the interfacial region with a relative amount that is about half that of bulk Bi_2_Se_3_. In addition, Bi is present at the top Au surface within a thickness range of less than 1 nm. This is attributed to the migration of Bi from the uppermost lattice planes of Bi_2_Se_3_ where Bi depletion is observed. Au migration toward Bi_2_Se_3_ is not observed, and neither Se nor Bi are detected to be present in the Au layer above the sensitivity limit of EELS.

**Figure 4:**
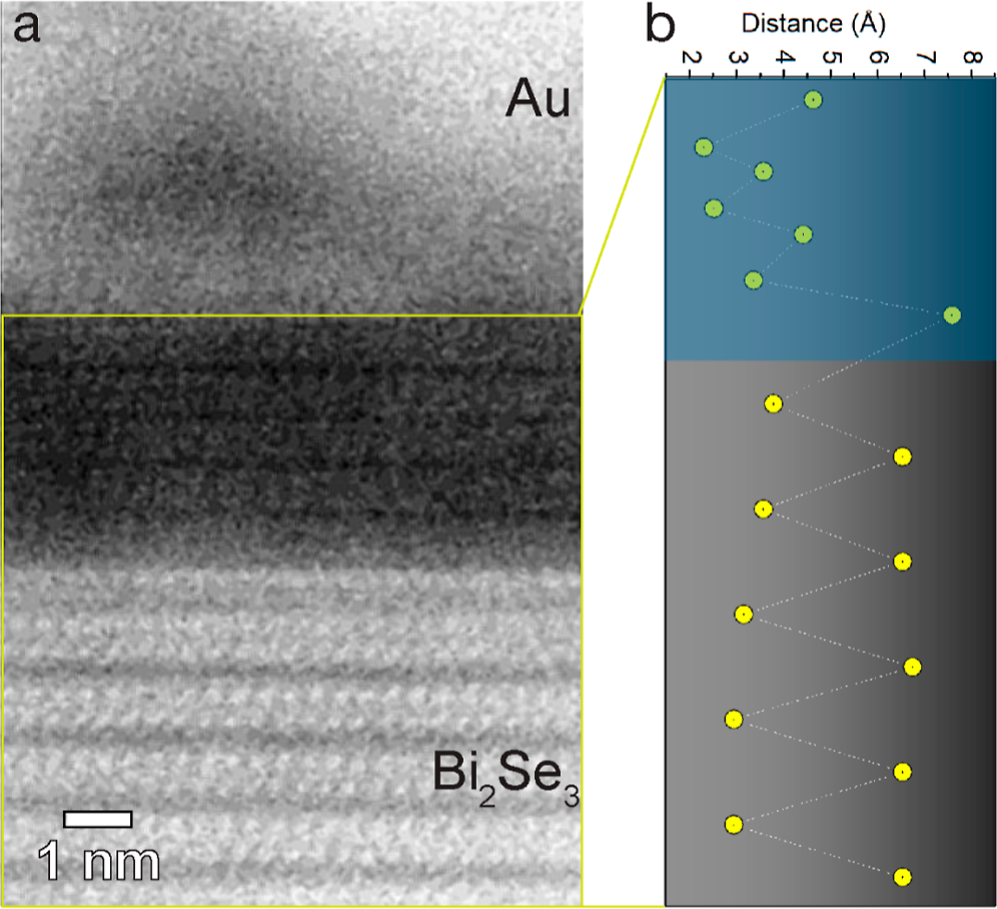
(a) HAADF/STEM image around the interfacial region analyzed in Figure 3c,d. (b) Measured vertical Bi–Bi plane spacing showing the collapse of the van der Waals spacing close to the interface with Au (blue shaded region).

Figure 4 shows a HAADF image of the thick interfacial region in Figure 3d. The interface is crystalline, with an atomic lattice resembling that of Bi_2_Se_3_. We analyzed the spacing between the Bi atomic planes along the vertical direction (Figure 4b), which shows a smaller spacing for the Bi–Bi within a quintuple layer in Bi_2_Se_3_ and a bigger distance for the Bi–Bi distance across the van der Waals gap (between two quintuple layers). As shown, the distance between Bi–Bi across the van der Waals gap is reduced to almost the Bi–Bi distance within the quintuple layer in the interfacial region, but the spacing modulation remains. These results suggest the formation of an intermetallic compound with a lower Bi content than Bi_2_Se_3_ at this interface. The phase diagram of Bi–Se only shows stable compounds with higher Bi content than Bi_2_Se_3_,^23,24^ unlike the Bi–Se compound experimentally observed here. Only a few Bi–Se compounds have been reported to have Bi atomic content below 40%, but the stoichiometry and structure are not compatible with the one observed here.^25^ Moreover, we also performed energy-dispersive X-ray spectroscopy to study the composition in larger areas and gain larger statistics, obtaining results equivalent to those obtained by EELS (data not shown). Both regions are found to extend for hundreds of nanometers, and in a few cases, they are observed to coexist next to each other.

**Figure 5:**
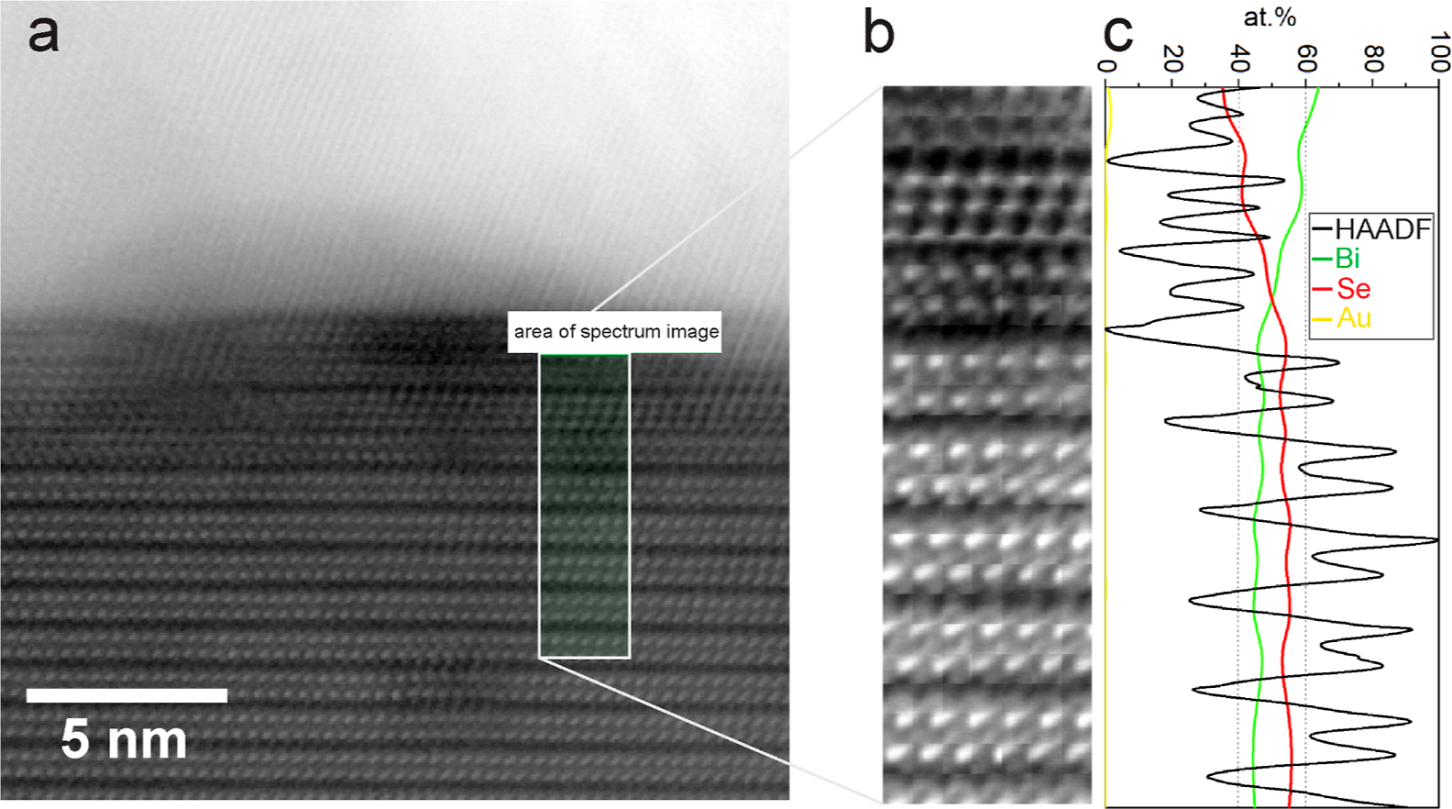
Post-mortem analysis after an in situ heating experiment that allowed the sample to heat to 200 °C before bringing it back to RT. (a) HAADF/STEM image and (b) zoomed area marked in (a) showing the structure at the Bi_2_Se_3_–Au interface with missing Se planes at the upper interface. (c) Vertical profiles of positions of the atomic spacings averages by the image in (b) and relative quantification of Se–L, Bi–M, and Au–M edges calculated from EEL spectra. The nominal composition of the bulk Bi_2_Se_3_ of Bi/Se 60:40 is well preserved away from the interface.

Further, we studied the role of higher temperatures on the reactivity between Au and Bi_2_Se_3_ by heating the material in situ in the TEM up to 200 °C for 1 h. The main observation was the loss of Se through the Au film and its accumulation onto the Au top surface (Figure S2), which resulted in the formation of a Se-deficient lattice at the uppermost Bi_2_Se_3_ planes (Figure 5). The Se deficiency is evident from the HAADF image (Figure 5a,b), where the Se-deficient region shows a crystalline structure very similar to that of Bi_2_Se_3_ but with the characteristic van der Waals gap of Bi_2_Se_3_ missing for some planes. EELS elemental maps (Figure 5c) confirm the reduced Se content.

Based on the described results, we can conclude that the interface between Au and Bi_2_Se_3_ is not stable at RT. We propose the following interaction mechanism. The interface strain, developing during Au deposition, is relaxed by a complex process involving a redox reaction between Bi^3+^ and Se^2–^ and subsequent diffusion of the resultant species through the Au layer. The redox reaction itself can be written as
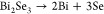
1

**Figure 6:**
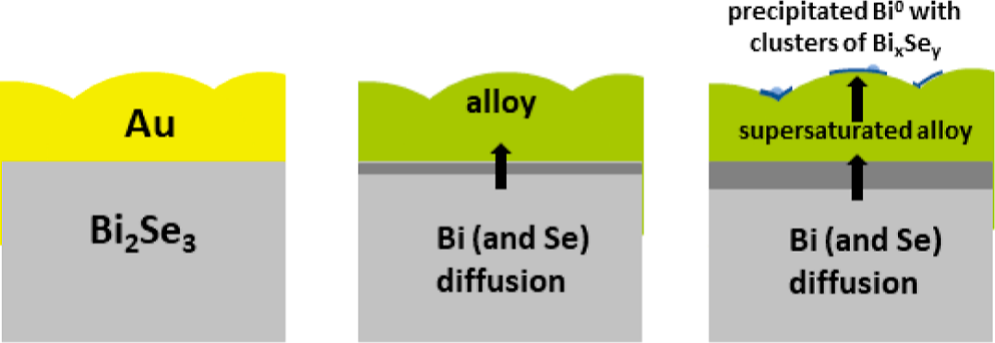
Schematics of the diffusion path of bismuth (and selenium) through the Au layer after the redox reaction (see eq 1) and formation of metallic species and Bi_*x*_Se_*y*_ clusters.

The formed metallic Bi dissolves in the Au layer, reaching a state of supersaturation. From such supersaturated alloy, bismuth heterogeneously precipitates at the top of the Au layer (Figure 6). The process resembles the mechanism known as the vapor–liquid–solid (VLS) method, wherein a vapor-phase reactant is introduced into a liquid droplet, leading to supersaturation and subsequent nucleation of a solid phase, mainly in the form of nanowires or nanorods.

Se also tends to follow the same diffusion process; however, the diffusion rate at RT is impeded due to the extremely limited solubility of Se in Au.^26,27^ This leads to an excess of Se in the Bi_2_Se_3_-side of the interface, resulting in the restructuring of the interface layers. The reduction in the charge of Bi and Se causes the collapse of the van der Waals gap and the formation of the intermetallic layer with a composition close to BiSe_3_. At the top of the Au layer, the diluted Se^0^ atoms are not stable and form Bi_*x*_Se_*y*_ clusters as seen by XPS. As the temperature rises, the diffusion of selenium becomes increasingly pronounced, leading to a notable deficiency of selenium in the interface region and subsequent structural reconstructions. This observation is consistent with a well-established fact about the exceptionally low evaporation energy and high vapor pressure of selenium compared to other metals.^28^

## Conclusions

Our study focused on the complex interactions at the interface between Bi_2_Se_3_ and deposited Au, with a particular emphasis on RT processes. Through our research, we have uncovered a multifaceted process involving redox reactions, diffusion, and structural transformations at this interface. This process resembles the vapor–liquid–solid mechanism, involving a redox reaction between Bi^3+^ and Se^2–^, followed by the diffusion of Bi^0^ through the Au layer and its subsequent precipitation onto the top of the Au layer in metallic form. Interestingly, we also observed a similar process, albeit at a slower rate, involving Se^0^, which on top of Au forms clusters with the surface-bound bismuth. The significantly slower rate of diffusion for Se and Bi species leads to nonstoichiometric conditions in the interfacial region. To compensate for this, structural changes in the interface layers occur, resulting in the formation of an intermetallic compound with a low Bi content. With an increase in the temperature, Se diffusion intensifies, leading to Se deficiency at the interfacial region and subsequent reconstructing of the interface. Overall, our study provides valuable insights into interface stability and composition, crucial for advancing material design and facilitating the development of robust TI-based devices.

## Supporting Information Available

Parameters of the Doniach–Sunjic function convoluted with the Gaussian function used to fit Se 3 d and Bi 5d core level spectra, view of the lamella on the heating chip and the finished lamella employed for heating, and HAADF image after the heating process (PDF)
