# Supplementary material for: Chemical Interactions at the Interface of Au on Bi2Se3 Topological Insulator
Source: J Phys Chem C Nanomater Interfaces. 2024 Sep 13;128(38):16154–60. doi: 10.1021/acs.jpcc.4c04241 (PMC11440592; doi:10.1021/acs.jpcc.4c04241)
Supplement: Supplementary file 1 — jp4c04241_si_001.pdf [file jp4c04241_si_001.pdf]

SUPPLEMENTARY

Chemical Interactions at the Interface of Au on Bi_2Se_3 Topological Insulator

Matjaz Valant,[1]* Sandra Gardonio,[1] Saul Estandia,[2] Mattia Fanetti,[1] Andrey Vladimirovich Matetskiy,[3] Polina Makarovna Sheverdyaeva,[3] Paolo Moras,[3] Vasiliki Tileli[2]

1 University of Nova Gorica, Vipavska 11, 5000 Nova Gorica, Slovenia

2 Institute of Materials, École Polytechnique Fédérale de Lausanne, CH–1015 Lausanne, Switzerland

3 CNR-Istituto di Struttura della Materia (CNR-ISM), SS 14, Km 163.5, 34149 Trieste, Italy

* corresponding author email: matjaz.valant@ung.si

Table S1: Parameters of the Doniach-Sunjic function convoluted with the Gaussian function used to fit (a) Se 3 d and (b) Bi 5d core level spectra reported in Fig. 2. In the fitting, spin-orbit splitting (SO) and core-hole lifetime (γ) were kept constants. For the fit of both Se 3d and Bi 5d core level spectra, it was necessary to use non-statistical branching. The deviation could be due to photoelectron diffraction effects. The intensity, energy and Gaussian widths (σ) of each doublet were considered as free parameters.

(a)

Au thickness	0 Å	110 Å	400 Å	1090 Å
**Se_1 (eV)**	**53.33**	**53.33**		
γ (eV)	0.08	0.08		
σ (eV)	0.62	0.7		
SO (eV)	0.74	0.76		
BR	1.88	1.6		
**Se_2 (eV)**		**53.45**	**53.42**	**53.53**
γ (eV)		0.08	0.08	0.08
σ (eV)		0.76	0.77	0.83
SO (eV)		0.76	0.78	0.78
BR		1.5	1.5	1.5

(b)

	0 Å	110 Å	400 Å	1090 Å
**Bi_1 (eV)**	**24.79**	**24.78**	**24.8**	**24.8**
γ (eV)	0.1	0.1	0.1	0.1
σ (eV)	0.65	0.82	1.8	1.82
SO (eV)	3.04	3.03	3.05	3.05
BR	2.19	1.83	1.6	1.6
**Bi_2 (eV)**		**24.2**	**24**	**24.1**
γ (eV)		0.08	0.1	0.1
σ (eV)		1.15	0.87	0.86
SO (eV)		3.05	3.05	3.05
BR		1.60	1.7	1.6

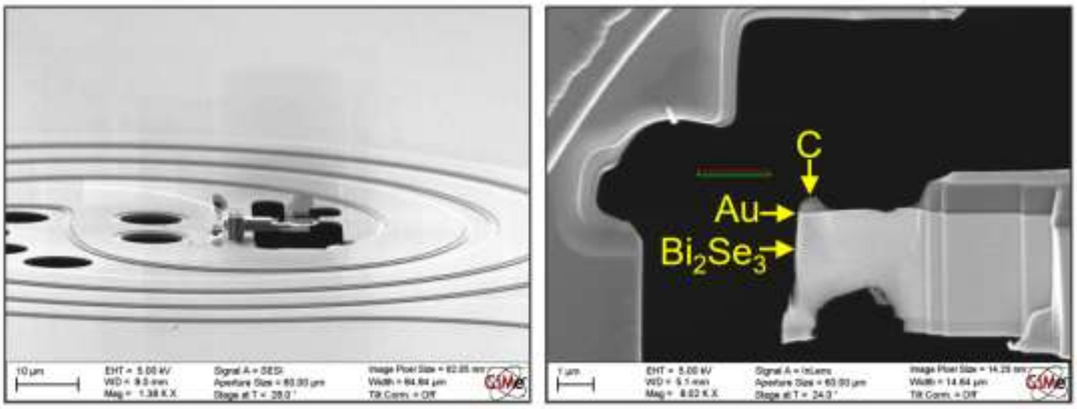

Figure S1. Left: view of the lamella on the heating chip. Right. View of the finished lamella employed for heating.

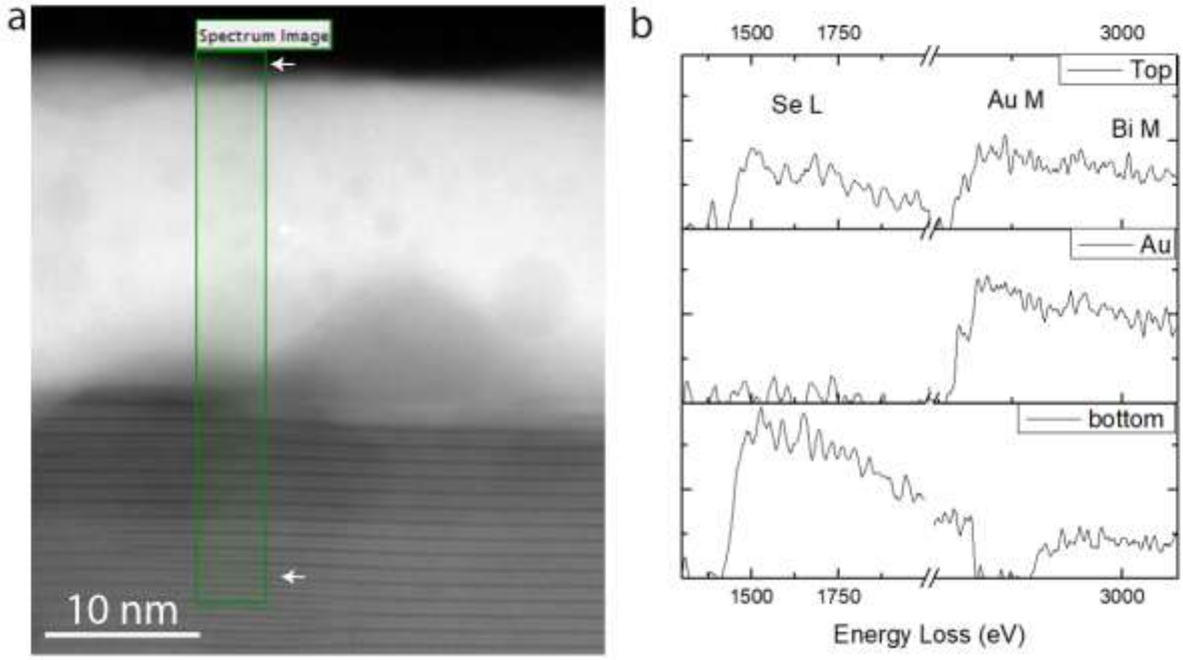

Figure S2. (a) HAADF image after the heating. (b) Se-L, Au-M and Bi-M edges at the locations indicated with arrows in (a).
